# Strengthened workplace relationships facilitate recovery at work – qualitative experiences of an intervention among employees in primary health care

**DOI:** 10.1186/s12875-021-01388-x

**Published:** 2021-03-09

**Authors:** Lina Ejlertsson, Bodil Heijbel, Ingemar H Andersson, Margareta Troein, Annika Brorsson

**Affiliations:** Department of Clinical Sciences, Family Medicine and Community Medicine, Lund University, Malmö, Sweden

**Keywords:** Recovery, Employee health, Primary health care, Intervention, Qualitative research, Health promotion

## Abstract

**Background:**

The literature on workplace interventions focusing on recovery is scarce; hence this study intends to expand that knowledge. An intervention was run for one year, aiming at increasing the experience of recovery during the workday among primary health care employees. During the intervention, different forms of recovery activities were integrated into the daily work at six primary health care centres. The aim of this study was to explore the intervention process and its effects.

**Methods:**

After completion of an intervention, 39 employees in seven focus groups were interviewed about their experiences of the intervention. A semi-structured interview guide was used, and the qualitative analysis was conducted by systematic text condensation.

**Results:**

Despite different conditions and attitudes when the project was launched, the participants portrayed a positive outcome of the intervention at all six workplaces. Four promoting factors for intervention success were identified: support, legitimacy, customization, and simplicity. Also, three areas of improvement during the intervention period were described: the workplace climate, employee well-being, and recovery awareness, which in turn became promoting factors.

**Conclusions:**

An intervention aiming at increasing workplace recovery can be promoted by support, legitimacy, customization, and simplicity. By using these promoting factors, health care workplaces can implement activity models which could increase employees’ experiences of recovery during the workday. Positive effects on workplace climate and employee well-being can also be achieved.

## Background

Health care staff are considered to be at high risk when it comes to stress-related disorders [1, 2], and primary health care in particular is highlighted as a setting for poor psychosocial work conditions. Several studies have shown a high level of stress and work demands, as well as negative effects on physical and mental health among primary health care employees [3, 4]. The opportunity to recover from these work demands is essential for the well-being of the employees [5–7]. Traditionally, work recovery research has focused on the importance of recovery outside work through, for example, psychological detachment from work [8] and sleep [9]. A recent study, where recovery during the workday was included, showed that recovery was the most important factor associated with employees’ self-rated health [10]. Hence, the workplace should be an important setting to promote recovery.

There are several examples of workplace interventions aiming at enhancing positive work experiences among health care staff, by reducing stress and promoting mental health and well-being [11, 12]. For example, an intervention addressing nursing staff effectively improved staff teamwork and work engagement by using discussion sessions, training in teamwork knowledge, and employee involvement [13], while another intervention reduced stress and increased the quality of life in health care professionals by using mindfulness-based stress reduction [14]. Also, an intervention study using gratitude, i.e. directing the health care practitioners’ attention to events they were thankful for at work, reduced stress and improved mental health [15].

Intervention studies focusing on recovery during the workday are limited. However, there are some examples. De Bloom et al. [16] introduced park walks and relaxation exercises during lunch breaks to promote recovery. The results showed that it may maintain and improve the well-being of employees, even if the effects were considered small and had a short duration. Yet another study has explored relaxation as a potential recovery activity, with focus on progressive muscle relaxation during the lunch break, where lower levels of strain were reported by the participants [17]. The effects of mindfulness during working hours for employee recovery have also been investigated previously, showing less emotional exhaustion and more job satisfaction in the intervention group [18]. Interventions could be a successful course of action for enhancing the well-being and recovery experience of employees. However, workplace interventions focusing merely on recovery, with a wider selection of recovery activities throughout the workday, are yet to be studied. Therefore, we earlier performed an intervention aiming at increasing employees’ recovery experience during the workday [19].

The aim of this qualitative study was to explore an intervention process and factors contributing to its effects. In addition, we wanted to study the participating employees’ perceptions of the intervention outcome, related to the individual and the workplace.

## Intervention

Based on earlier studies, showing that recovery was the most important factor for employee health [10] and that variation, companionship, and manageability were of importance for recovery [20], an intervention study was planned.

The intervention, which had a salutogenic, i.e. health promoting perspective [21], took place in the primary health care work context in Sweden. All employees of different professional groups participated, including nurses, physicians, paramedical staff, and administrative staff. Recovery models for each of the six participating primary health care centres (PHCCs) were developed, based on the areas of variation, companionship, and manageability and modified according to the PHCCs’ own abilities, needs, and wishes. Established methods for decreasing work-related fatigue and stress, combined with increasing well-being and recovery, were also considered. The intervention was run for one year and started off with each centre forming a small group of employees from various professions, so called inspiration group. This group – together with the researchers – were responsible for generating, elaborating, and implementing ideas about how recovery could be integrated into the daily work at their workplaces. All employees at each of the participating PHCCs also had the opportunity to contribute to the development of the intervention model throughout the intervention period. Six intervention models were introduced, one at each participating workplace, with different types of recovery promoting activities (Table 1). The activities could either be carried out individually, together with co-workers, or with the whole employee group. The individuals decided for themselves which activities they wanted to perform, when, and how.

**Table 1:** Description of activities implemented at the PHCCs

Recovery activities done individually or together with co-workers	Recovery activities together with the whole employee group
Deep breathing exercises	Recovery reminders in the form of coloured stickers around the centre
Relaxation exercises	Monthly recovery reflection
Stretching exercises	Notice board with positive messages
Access to relaxation room	Team building activities
Interprofessional reflection group meetings	Morning meetings
Access to gym	Organized after-work activities
Lunch break walks	Joint physical activity exercises
Mail with mindfulness exercises	Concept discussions (e.g. manageability, influence, companionship, feedback)
Brief positive messages on toilet door	Weekly positivity letter from manager
	Mindfulness sessions
	Joint breakfasts
	Workplace development day
	Changes in the physical environment
	Management team
	Music in the break room
	Suggestion box for recovery ideas
	Reflection sessions
	Breakroom as a work-free zone
	Step counter contests
	Medical yoga therapy
	Basic body awareness therapy

The results of the intervention were evaluated by questionnaires to the participating employees, before and after the intervention [19]. The quantitative evaluation revealed a significant increase in experienced recovery during the workday, after one year of intervention.

## Method

### Setting and participants

After the intervention, each of the six intervention PHCCs were asked to enrol a group of voluntary employees as participants in a focus group interview. Five focus group interviews with employees – not part of the inspiration groups – were conducted, while one of the employee groups declined participation. Two focus group interviews with mixed members of the six inspiration groups were also carried out. All the interview groups consisted of four to seven participants with different professional backgrounds. Altogether, 39 employees, 34 women and five men, took part in the seven focus groups (Table 2).

**Table 2:** Description of focus group participants according to sex and profession

	*n*
**Sex**	
Women	34
Men	5
**Profession**	
Nurse	19
Physician	1
Paramedical staff	9
Administrative staff	10
Total	39

### Data collection

A qualitative approach with focus groups was considered appropriate to explore the variety, and to get a deeper understanding of the experiences, perceptions, and opinions of the participants. Two of the authors conducted the focus group interviews, one of whom had the role of moderator and the other acted as an observer. All interviews took place in an undisturbed room at the PHCCs, either at the participants’ own centre or, for the mixed groups, at a nearby centre. A semi-structured interview guide was used to encourage a dialogue between the participants, with open-ended questions like:”What was your experience of the intervention?” and “In what way has the intervention affected your experience of recovery during the workday?”. The participants were also asked to describe promoting and limiting factors linked to the intervention and its activities. Depending on the discussion, clarifying questions could be asked to get a deeper understanding of the participants’ experiences of the intervention process. All focus group discussions lasted for approximately one and a half hours. The interviews were recorded with a digital voice recorder and transcribed verbatim by an experienced transcriber.

### Analysis

The analysis, with an inductive approach, was inspired by systematic text condensation according to Malterud [22]. Initially, the entire transcripts were read separately and repeatedly by three of the authors, in order to get an overview of the data. After that, preliminary themes were identified and content according to these was marked. Thereafter, smaller text units with similar substances were identified and labelled with codes. The codes were then merged into broader categories and subcategories, which were then compared to the original data. The researchers met on several occasions, and all subcategories and categories were discussed and adjusted until consensus was achieved. A confirmatory reading of transcripts in relation to subcategories and categories was made by the remaining two authors. All quotations in the results section are identified by a number after the quotation, denoting which interview it belonged to.

## Results

The participants reflected on how their initial working conditions affected both how the intervention was met when first being introduced and how the intervention was designed. These conditions concerned leadership, such as accessibility or exchange of managers, as well as staffing, workload, workplace structure, and physical environment. Also, the psychosocial environment played an important role, with focus on workplace relationships.

Regarding how the intervention was initially received, four types of attitudes were noticed by the researchers and confirmed in the focus group interviews. On the one hand, there were workplaces with a strong starting position, with the first one saying “We are already doing well. It can always get better”, in contrast to the second one saying “We are already doing well. We do not need this”. On the other hand there were workplaces with a more challenging starting point where the reactions were “We are having a tough time right now. We need this”, versus “We have a tough time right now. Focusing on recovery feels like a mockery”. Regardless of these initial conditions and attitudes, a positive outcome of the intervention was reported by the participants in all focus groups.

Four promoting factors enabling the intervention process, which enhanced the employees’ recovery experience during the workday, were identified. These were the categories titled support, legitimacy, customization, and simplicity (Table 3).

**Table 3:** Overview of promoting factors

Categories	Subcategories
Support	Help
	Feedback
	Encouragement
Legitimacy	Transparency
	Ownership
	Enthusiasm
Customization	Flexibility
	Maintenance
Simplicity	Convenience
	Integration

In addition to the promoting factors, several areas beside the recovery experience improved during the intervention process. This resulted in three categories called upward spirals: climate, well-being, and awareness (Table 4). The stronger the upward spirals became, the more they had a positive influence on the intervention and the employees’ experience of recovery. Hence, the upward spirals became both outcomes and promoting factors.

**Table 4:** Overview of upward spirals

Categories	Subcategories
Climate	Companionship
	Familiarity
	Openness
	Influence
Well-being	Calm
	Joy
	Energy
	Exercise
Awareness	Eye-opening
	Ripple effect

### Promoting factors

#### Support

The participants explained how the support from co-workers, as well as managers, was an important part of how well they managed to enhance their recovery experience. The three subcategories were: help, feedback, and encouragement.

*Help* was the first subcategory, where it became clear that it makes it easier if you have someone that can cover for you while you get a cup of coffee or some fresh air when needed. Likewise, someone to collaborate with in trying to reduce the workload, which in turn will facilitate breathing spaces throughout the workday.

*… I think that when we’re all friends and we help each other and you get flow in the work, that you get help and give help and that kind of thing, then you get to recover when you feel that it’s quite okay for me to go out and sit down for ten minutes and have a cup of coffee in the morning …* (2).

The second subcategory was *feedback,* where the participants explained how it pushed them a little further when they got positive feedback on the recovery activities that they performed. When someone told them that they saw what they did, and that they did well, the satisfaction increased the likelihood of doing it again.

*But positive feedback, I think, actually gives a lot. When you give feedback to a co-worker who did something good.*

*Yes… It strengthens you.* (3).

The third subcategory was *encouragement.* A key for success was the fact that many of the activities were performed together with others. The participants described how they encouraged each other to try various activities, and how positive cheers while executing an activity was a promoting element for doing it again. Also, the employees in the inspiration groups, who helped to plan and implement activities, explained how the appreciation from their co-workers made them willing to continue.

*… so you probably need to get a little more encouragement from time to time to get on or kind of keep it alive, so to speak …* (3).

#### Legitimacy

Legitimacy is about being given the mandate to act, but also about the personal commitment. In addition, legitimacy concerns the importance of being a workplace where there is a permissive attitude from both manager and co-workers when it comes to performing recovery activities. The three subcategories were transparency, ownership, and enthusiasm.

The first subcategory was *transparency*, where the participants talked about information as an essential factor. The information could be about the recovery activities, as well as information about what is acceptable at their workplace. Also, time for recovery must be set aside.

*I think one of the most important things is simply to inform about what opportunities there are for recovery, what suggestions you have, and then really emphasize that it’s okay to use these tools … that it’s okay to go away and use these tools for recovery, so that you can then come back and maybe be able to do a better job.* (6).

*Ownership* was the second subcategory, which describes the employees’ own responsibility for taking action and carrying through activities that promote their recovery experience. Only the participants themselves know what they need to feel recovered.

*So it’s us, it’s we ourselves who have this morning meeting … it’s perfectly obvious that we ourselves influence it, that you remember that, so to speak.* (3).

The third subcategory is called *enthusiasm.* The participants described how a group or project needs people who are dedicated and want to push forward. This does not only apply to the inspiration group, which was assigned a responsibility, but also to other employees who wanted to be an inspiration for others, i.e. driving spirits.

*But sometimes it may be enough that there’s like someone who’s a bit more go-ahead and a bit extrovert in a group, who can maybe involve … well, those who might find it a bit harder and are maybe a bit slower at the beginning and it can often be a bit liberating, so I think that as long as there are just a few heads that have a little more of that, I think it kind of brings the whole group along.* (4).

#### Customization

The recovery models were customized, which means that the activity range was adjusted to each PHCC’s own abilities, needs, and wishes. Hence, the intervention offered different activities for every employee to choose from. It was also important for the activities to be adaptable and continuously maintained since the conditions can change over time. The two subcategories were flexibility and maintenance.

*Flexibility* was the first subcategory. The content of the activity, as well as the time and place for the activity, needed to be adjustable in order to succeed. If the participants encountered any problems along the way, they quickly learned how to be flexible and move forward.

*And then it is a matter of being a bit quick-thinking and thinking about, can we change it in some way?* (6).

The second subcategory was *maintenance*, described as a way to sustain the activities. For example, new materials were put on the notice boards each week. Also, the participants knew what they needed to do to experience recovery but following it through was promoted via reminders of different kind. These could be oral, or reminders in the form of coloured stickers around the centres.

*Somehow you need to get these reminders, I think, continuously.* (3)

#### Simplicity

The participants stated that a distinct promoting factor was that the activities were simple to carry out. There should not be too many obstacles in the way, because it might lead to employees not participating. The two subcategories were convenience and integration.

*Convenience* was the first subcategory, which explains how the activities should be formed. The activities should be simple to understand and not take much time to carry out, so that they did not interfere with the participants’ ordinary routines. Also, the participants talked about how activities that did not require much effort on their part were especially appealing, where they could just turn up and “go with the flow”.

*… it was so very simple and smooth and doesn’t take so long …* (7).

The second subcategory was *integration,* which describes how some of the activities became a natural part of the workday. This, in turn, increased the chances of carrying out the activities.

*And that you get used to things that make it come naturally.* (2).

### Upward spirals

#### Climate

The participants found that the workplace climate became more positive during the course of the intervention. The positive climate was described as a feeling of mutual concern for each other, where everyone felt involved and could collaborate as professionals as well as laugh together as friends. The four subcategories were companionship, familiarity, openness, and influence.

The first subcategory was *companionship*. The employees highlighted that both professional relationships and friendships had been strengthened over the year. They said that the important thing is not what they do, but that they do it together. An activity that contributed to this feeling was the morning meetings, but also team building activities and the fact that many of the activities were carried out together with others.

*… I imagine that … that it [the project] strengthens the cohesion and the sense of solidarity…* (5).

The second subcategory of climate was *familiarity*, where the participants explained that the understanding of each other’s work situation and person had increased during the intervention. Among other things, this was strengthened by “get to know your co-worker” messages on the notice boards and after-work activities, which promoted conversations between staff members.

*And I think that no matter what task you have, you’re… when we are here, we are both our profession and our person, you can’t just separate them, somehow. So it’s nice to have some insight into the other as well.* (5).

*Openness* was the third subcategory, which describes a more open-minded climate where all members of the employee group are seen and listened to. A contributing factor was the reflection sessions where everyone got to speak their mind about their work experiences.

*So ….yes, but positive, I think it actually contributed a little bit to kind of open up a little bit, to create a little … a somewhat lighter climate as well. I suppose that’s how I feel it.* (4).

The fourth subcategory was *influence*. The participants experienced an increased influence, regarding the workplace structure and the intervention progress, by participation in various enhancing activities. For example, group discussions during the workplace development day and the introduction of suggestion boxes for workplace recovery ideas.

*If you feel that you have very little influence, even if you understand that this has to be implemented because it has been decided, but if you feel that you don’t have much influence, that you can still in some way influence it, then you take this in a completely different way.* (2).

#### Well-being

The employees experienced improved well-being during the year of the intervention, describing various positive emotions such as being happier and more relaxed or alert, depending on the context. The four subcategories were calm, joy, energy, and exercise.

*Calm* was the first subcategory, where the employees expressed that relaxation exercises or listening to music made them calmer. Designating the breakroom as a work-free zone had a similar effect. They described how good it felt to use some of the recovery activities to unwind when their stress level was high.

*Well, you still felt relaxed in a way, so … you felt relaxed when you got up and started working afterwards.* (4).

The second subcategory was *joy*, which was described as an increase in laughter and having fun together as a group. The participants depicted how doing things together contributed to this joy. Some examples were step-counting contests and joint breakfasts. Also, the notice boards with positive messages acted as a place for gathering.

*… but it’s a different kind of good atmosphere now, and a whole new joy … it’s almost as if it’s built into the place now. Before, it was that you kind of felt it like this, but now it kind of pervades everything, in my experience.*

*Yes, you kind of get a more positive feeling.*

*That you maybe feel better than you have done before.* (4).

*… it’s recovery when you hear laughter.* (5)

The third subcategory of well-being was *energy*. The participants portrayed some of the activities as “a good start to the day”, “mind clearing”, and a way to increase their focus, with mindfulness sessions and lunch break walks as two examples of this kind of energizing activities.

*… then I felt kind of alert and fit for fight again, and both the patients and the colleagues benefit from that …* (3).

The fourth subcategory was *exercise*, where the participants explained how the intervention contributed to an increased level of physical activity and a feeling of being healthy. Two activity examples were the gym access and the joint physical activities, such as medical yoga therapy or workout exercises.

*Well, it feels good, you know, I feel good when I’m working out and then everything gets better, like, and I feel better … meeting the patients in it, so I think it’s really nice when you have the opportunity to do it.* (4).

#### Awareness

During the one-year intervention, the awareness increased in several ways. The employees explained that they noticed new things regarding their experience of recovery, as well as how what they do can affect others. The two subcategories were eye-opening and ripple effect.

The first subcategory was *eye-opening*, where the participants described how the project had made them aware of the concept of recovery and what it meant to them. The concept discussions and monthly recovery reflection were pointed out as particularly fruitful.

*I think I have become aware that there are many different forms of recovery, just that people think recovery is different things, whether it’s going away or if there is a funny sticker or if there is better structure in the work maybe.* (6).

*Ripple effect* was the second subcategory, which described how small changes or attempts at recovery influenced other activities. For example, a stretching exercise performed by one employee could inspire co-workers to try the same. Or they could have after-work activities, which then had positive effects on their everyday work in terms of building relationships.

*… I put up and change a bit on the inspiration board, because it gives me something and I hope it will spread ripples when you do something like that.* (7).

## Discussion

### Principal findings

Four factors for promoting a successful workplace intervention were described by the participants in the focus group interviews. These were support, legitimacy, customization, and simplicity. Also, three areas were positively affected by the intervention. These upward spirals were a better work climate, improved well-being, and increased recovery awareness among the employees, which in turn further promoted the progress of the intervention, i.e. they became promoting factors. An increase in perceived recovery during the workday was described regardless of the PHCCs’ initial conditions and the attitudes of the participants towards the introduction of the intervention. This is in line with the results in the quantitative evaluation of this study [19], which showed an enhanced recovery experience during the workday in the intervention group.

### Findings compared to other studies and literature

Companionship, with all its nuances, was an underlying element in the data, which can be seen in both the promoting factors (e.g. support) and the upward spirals (e.g. climate). Hence, strong workplace relationships can promote the implementation of recovery activities, but also be further strengthened by the activities themselves. In a previous qualitative study [20], companionship was identified as one of the key influencing factors for experiencing recovery during the workday. Also, a literature review on subjective well-being highlighted supportive social relationships as a vital predictor of well-being [23]. Social relationships at the workplace have been shown to be an essential determinant for experiencing positive emotions at work [24, 25]. To experience belonging with co-workers, with a feeling of being valued, respected, and accepted by others at the workplace, is an essential factor for employee well-being [26]. Belonging is therefore seen as an important resource for workplace health promotion, which has been illustrated in several qualitative studies [27, 28]. This was also described by the participants in the present study when they discussed the increased openness and familiarity that came with the intervention, with the help of some of the recovery activities that were performed. A Swedish study on health care workers found that employees who experienced a good atmosphere, feedback, and meaningfulness in work also reported a better health [29]. They concluded that employees’ self-rated health is associated with their experience of positive workplace relationships with both co-workers and managers. The importance of positive feedback and encouragement from the working group also emerged from the current interviews, where they were described as promoting factors for performing recovery enhancing activities during the workday.

A factor that permeated the intervention process and its positive outcome was communication. To communicate accurate information, as well as reminders, about the activities was mentioned by the participants as a promoting factor for the success of the intervention. Dickson-Swift and colleagues [30] stated that good communication, including information, workplace structure, and friendly co-worker interactions, is a fundamental element for creating a healthy working environment. Social chat between co-workers was encouraged through several activities in the current study, which led to an increased feeling of joy and familiarity. Thanks to the project, employees started to communicate about recovery. A high degree of awareness in an individual has been shown to increase the chance of a long-term behaviour change [31]. In this study, the awareness was described by the participants as eye-opening and an important factor for the intervention to function.

Legitimacy was mentioned as an essential factor for enabling the performance of recovery activities. There was a demand for transparency at the workplace, stating the time accessible for recovering activities. There was also a need for ownership, in which the employees themselves took responsibility for performing the activities. In a study on work breaks, the researchers concluded that autonomy is a key factor for well-being [32]. They suggested that the possibility for employees to choose their own activity during breaks is as important as what they actually do during the break. Also, in a qualitative study exploring why general practitioners remain at their jobs, the room for autonomy and independence was emphasised [33]. In our study, the feeling of having influence was important and one of the areas of improvement during the intervention period. The employees had the opportunity to decide which activities should be implemented at their workplace. Also, some of the activities that were performed had a focus on enhancing the employees’ experience of influence over their work situation. In a study on indicators for a healthy work environment at primary care units, the employee involvement and presence of team spirit for reaching joint goals were described as promoting factors [34]. This coheres with the current result showing that employees being enthusiasts, i.e. able to inspire others and drive the project forward, were important when striving at a successful intervention outcome.

Customization, which focuses on the specific needs of the employee group and the workplace, was recognized as a promoting factor for this intervention. Similar findings were presented in a review on interventions promoting mental health and happiness among health care workers [11], were customization was an important theme for intervention success. Their second theme was the commitment of the employees, which is also in line with our results. We saw that employee participation and engagement often led to a ripple effect which further helped sustaining the activities and their positive effects on the individual, the work group, and the workplace. The aspect of maintenance is also mentioned in the review [11], in a theme regarding the longevity of an intervention and the effect on employees’ health. In addition to maintaining the recovery activities, we endeavoured to integrate the activities into daily work, making them as quick and easy as possible for the convenience of the participants. These factors all helped to facilitate successful implementation of the recovery activities and to promote the positive outcome of the intervention.

Irrespective of the different – and constantly changing – conditions, all the PHCCs in our study seem to have benefitted from the intervention. This result was described by the participants in the focus groups and shown in the previous questionnaire study evaluating the intervention [19]. Complexity is mentioned as the last theme in the review of workplace interventions [11]. This refers to the challenge when, for example, organizational changes occur at the same time as the intervention. The difficulty in implementing an intervention that will suit a whole group of employees, regardless of their different needs, is also highlighted. The customization of the intervention, together with being flexible and adaptable over time, were reasons why those obstacles were overcome, and a favourable outcome was achieved in our study.

The well-being of the participants in this intervention improved during the year, and experiencing more energy was an important component. The employees also expressed that they felt more joy, were calmer, and had increased their levels of physical activity. A study of the concept of energy [35] showed that it is possible to have an energy-building experience at work, i.e. feeling that the work gives energy and that the energy received is greater than the energy expended. It was confirmed that the strongest predictor for having an energy-building experience was to also experience recovery, followed by experiencing joy when coming to work. The results in our study correspond with existing evidence suggesting that employees can improve their well-being by participating in workplace interventions involving physical activities such as taking walks and practising yoga [36]. Also, using mindfulness-based stress reduction for enhancing psychological functioning such as relaxation and self-compassion [37].

## Strengths and limitations of the study

A strength in this study is that the intervention process and its effects were reflected on from two different perspectives, by including both members and non-members of the inspiration groups in the focus group interviews. Moreover, managers and owners were not included in the focus groups, as an attempt to reduce the risk of the employees feeling inhibited to share their experiences. However, we acknowledge the possible limitation of not capturing the managers’ and owners’ experiences of the intervention.

A strength is that the five authors have different background experiences in both public health and general practice, which comes with various perspectives and pre-understandings, which is important when conducting the data collection, the analysis, and the writing of this article.

A limitation could be that the participants’ memory may have revised how they experienced the intervention. Nevertheless, the risk can be considered small because the interviews were conducted between two and four months after intervention ending, which is a relatively short period of time. Also, several recovery activities were successfully integrated into daily work, which made them ongoing during the time of the interviews. This can likewise be favouring with regards to the participants’ memories of the activity experiences.

We acknowledge the possible Hawthorne effect [38], i.e. a change in the participants’ behaviour as a response to the attention generated by the project, and not because of the intervention itself. Another limitation is the risk of not receiving the experiences of all employees working at the participating PHCCs, but only those who volunteered to take part in the focus group interviews. Though, this might have led to the engaged and experienced employees signing up for participation, which can be considered a strength with regards to the content of the collected data.

## Conclusion

This study attempts to contribute to the hitherto scarce research on interventions promoting recovery at work. More specifically, this means developing the knowledge on how to use a workplace intervention to integrate recovery into daily work. By using the promoting factors support, legitimacy, customization, and simplicity, health care workplaces can implement activity models which could increase the employees’ experiences of recovery during the workday. Positive effects on workplace climate and employee well-being can also be achieved. The next step will be to compose a practical guide with more explicit directions on how to develop workplace interventions focusing on recovery.

## Data Availability

The dataset generated and analyzed during the current study are not publicly available due to the risk of individual privacy being compromised, but are available from the corresponding author on reasonable request.

## Abbreviations

PHCC: Primary health care centre
